# In Vitro Study of the Effects of *Angiostrongylus cantonensis* Larvae Extracts on Apoptosis and Dysfunction in the Blood-Brain Barrier (BBB)

**DOI:** 10.1371/journal.pone.0032161

**Published:** 2012-02-29

**Authors:** Xin Hu, Jiang-Hui Li, Lan Lan, Fei-Fei Wu, Er-Peng Zhang, Zeng-Mei Song, Hui-Cong Huang, Fang-Jun Luo, Chang-Wang Pan, Feng Tan

**Affiliations:** 1 School of Medical Laboratory Science and School of Life Science, Wenzhou Medical College, Wenzhou, Zhejiang, People's Republic of China; 2 Institution of Health and Environment, Wenzhou Medical College, Wenzhou, Zhejiang, People's Republic of China; 3 Department of Parasitology, School of Basic Medical Sciences, Wenzhou Medical College, Wenzhou, Zhejiang, People's Republic of China; 4 Department of Clinical Laboratory, Zhuji People's Hospital, Zhuji, Zhejiang, People's Republic of China; Washington University, United States of America

## Abstract

It has been hypothesized that blood-brain barrier (BBB) dysfunction in *Angiostrongylus cantonensis* infection might be due to the apoptosis of the hosts' BBB cells. Here, we evaluated this hypothesis through several methods, all based on an *in vitro* mouse BBB model consisting of primary culture brain microvascular endothelial cells (BMECs) and brain astrocytic cells (BACs). In the present study, a four-hour percolation and HRP permeability experiment showed that *A. cantonensis* larvae extracts can increase the permeability of the BBB. Apoptosis among BMECs and BACs after exposure to larvae extracts was monitored by TUNEL and annexin-V-FITC/PI double staining. *A. cantonensis* larvae extracts were found to induce apoptosis in both BMECs and BACs. For this reason, we concluded that the induction of apoptosis might participate in the BBB dysfunction observed during angiostrongyliasis. Improved fundamental understanding of how *A. cantonensis* induces apoptosis may lead to new approaches to the treatment or prevention of this parasitic disease.

## Introduction

The rat longhorn, *Angiostrongylus cantonensis*, is a parasitic nematode and one of the most common causes of eosinophilic meningitis in Southeast Asia and the Pacific. Rats serve as the definitive host of this nematode. A nonpermissive host, such as a human or mouse, could become infected by eating snails or uncooked vegetables containing the infectious third stage larvae of *A. cantonensis*. Afterward, the development of the parasites will terminate in the brain at the young–adult worm stage, causing eosinophilic meningitis or meningoencephalitis [1].

The blood–brain barrier (BBB) protects the central nervous system (CNS) from invasive agents, such as inflammatory cells, bacteria, and chemical agents. It is believed, therefore, that some dysfunction in the blood-brain barrier (BBB) is required for this parasitic disease to take hold in the human brain. This phenomenon has been recorded in the case of certain types of meningitis, cerebral malaria, and other CNS infections, all of which are characterized by the presence of leukocytes in the cerebrospinal fluid [2]–[6]. However, little is known about BBB impairment in parasitic helminth infections.

In a mouse animal model of eosinophilic meningitis caused by *A. cantonensis* infection, researchers have found that the BBBs of mice infected with *A. cantonensis* become impaired, as shown by the high concentrations of protein and albumin and by the high leukocyte counts that can be detected in the CSF [7]. Infection of the CSF typically causes severe inflammatory reactions, which are mediated by pathogen products and host cytokines. This inflammatory reaction compromises the function of the BBB, allowing vasogenic brain edema to develop. This edema in turn contributes to cerebral dysfunction and can worsen brain damage [8], [9].

Although *A. cantonensis* infection is known to be associated with lesions in the BBB, the issue of whether parasites can cause these lesions or other BBB impairments directly or whether they only take advantage of preexisting lesions has yet to be resolved. One study showed unidentified apoptotic cells in the brains of mice infected with *T. brucei* [10].

The types of cells involved in the BBB include microglia and endothelial cells. These cells have macrophage-related functions and are involved in inflammatory processes in the central nervous system, in part by preserving the integrity of the CNS and participating in the BBB in the perivascular area [11]. Apoptosis of these endothelial cells can change the endothelial layers necessary to the BBB and initiate inflammation. This may be related to the lesions observed during *A. cantonensis* infection.

One could then hypothesize that the BBB dysfunction observed in *A. cantonensis* infection may be caused by apoptosis of the brain cells of nonpermissive hosts. Dysfunctional apoptosis is involved in several infectious diseases [12]. Parasites may excrete certain factors that can induce or inhibit apoptosis in brain tissue cells, an adaptation that allows the parasite easier entry into the brain. Some of our own previous studies, using an ICR mouse angiostrongyliasis model, have indicated that *A. cantonensis* infection of mouse brains causes apoptosis in mouse brain tissue. The current study, continuing along this line, constructed a BBB cocultured model of ICR mice *in vitro*, and observed apoptosis induction and permeability of BBB model using *A. cantonensis* larvae extracts.

## Materials and Methods

### Ethics statement

Protocols involving the use of animals were approved by the Wenzhou Medical College Animal Policy and Welfare Committee (Permit Number: wydw2009-0001).

### Preparation of larvae extracts

Four-week old imprinting control region (ICR) mice (The Experimental Animal Center, Wenzhou Medical College, Wenzhou, Zhejiang, China) were fed *Ampullaria gigas spix* containing infectious third stage larvae of *A. cantonensis*. The mice were killed on day 4, and brain tissue was collected.

One thousand *A. cantonensis* young–adult worm stage larvae were collected from mice brain tissue under dissecting microscope and washed twice with 1 ml PBS containing 1 mol/l PMSF and then fully ground in liquid nitrogen. They were then homogenized and frozen and thawed five times. Worm suspension was centrifuged at 10,000×g for 5 minutes, and the supernatant was removed for use as larval extract. The extracts were filtered through a 0.22 µm millipore filter and the protein concentration of the extracts was measured by bicinchoninic acid (BCA) assay.

### Construction of *in vitro* mouse cell model of the blood-brain barrier (BBB)

In concept, the *in vitro* cell model of the BBB was built by culturing brain microvascular endothelial cells (BMECs) on the tops of Transwell inserts and brain astrocytic cells (BACs) on the bottoms. The astrocytic foot processes would make contact with the BMECs through the millipores in the Transwell filter membrane and promote the characteristic formation of a BBB. In practice, the mouse BBB cell model was built *in vitro* according to the following procedures established in our laboratory [13].

In order to obtain purified BACs, a primary culture of BACs was established. First, the cerebral cortices of 10 ICR mice were collected under sterile conditions, digested in 0.25% trypsin (Sigma, Saint Louis, MO, U.S.) at 37°C for 30 minutes, and then the digestion was stopped by the addition of BAC-specific Dulbecco's modified Eagle's medium (DMEM) (10% FBS, 100 U/ml penicillin, 100 U/ml streptomycin). The mixture was washed and filtered through 200-mesh sieve. Then the filtrate was seeded in a cell culture flask and placed in an incubator containing 5% CO_2_ at 37°C until cells covered the bottom of the flask. The medium was replaced by fresh BAC-specific medium. Cells were shaking-cultured at 37°C and 200 rpm for 15 hours. Loosely adherent cells were discarded and the collected purified BACs were identified by the astrocytic marker, glial fibrillary acidic portein (GFAP), using immunohistochemical staining. The positive rate of the BACs used in the experiments was always higher than 95%, as measured by GFAP staining. The purified BACs (2×10^5^/ml), afterward, were seeded on the bottom side of a Transwell filter (Milipore, Billerica, MA, U.S.) membrane. After 4 hours, the Transwell was placed upright, 850 µl of BACs-specific medium was added to the receiver tank (outside room) of the culture tank, and 650 µl of BACs-specific medium (20% FBS, 100 mg/l heparin sodium, 5 ng/ml FGF, 100 U/ml penicillin, 100 U/ml streptomycin) was added to the donor tank (inner room). Media were changed every other day.

In order to obtain purified BMECs, capillary fragments from the cerebral cortices of 10 ICR mice were isolated at 4°C, followed by digestion in 0.2% type II collagenase (Sigma) at 37°C for 30 minutes. BAC-specific medium was added and the cell suspension was seeded in a 24-well plate. The differential adhesion method was used to remove fibroblasts and obtain purified BMECs. Then, the purified BMECs were identified by the endothelial marker, factor VIII-related antigen, using immunohistochemical staining. The positive rate of the BMECs used in the experiments was always higher than 95%, as measured by factor VIII-related antigen staining. On the 5th day of BACs culture, after digestion, the purified BMECs were adjusted to 3×10^5^/ml and seeded at the top of the Transwell filter membrane. BAC-specific medium was added to the receiver tank and the donor tank. The fluid levels were kept the same on both sides and the fluids were changed every other day. When both types of cells started to grow in single layers (on about day 7), the *in vitro* BBB cell model was considered to have been successfully established.

### Determination of the action concentration of the larvae extracts

After the purified BMECs and BACs were digested, cell concentrations were adjusted to 1×10^4^ cells/well and the cell suspensions were added to 96-well cell culture plates. The plates were placed in a CO_2_ incubator for 24 hours. Then the media were replaced with serum-free cell culture media and the cell cultures were incubated for another 24 hours. Then 10 µg/ml, 20 µg/ml, 30 µg/ml, 40 µg/ml, 50 µg/ml, 60 µg/ml, 70 µg/ml, and 80 µg/ml of larvae extract was added to the BMEC and BAC culture plates at 100 µl/well for an additional 12 hours. CCK-8 reagent (Dojindo Laboratories; Kumamoto, Japan) was added to the 10 µl/well and the cells were cultured for another 4 hours at 37°C. The absorbance at 450 nm was assessed by ELISA. For each group, three duplication wells were given 100 µl/well PBS. These wells served as negative controls. The ability of different concentrations of larvae extracts to inhibit cell growth was calculated according to the following formula:
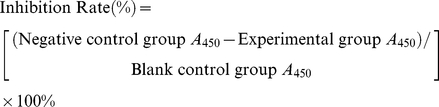



An inhibition rate curve was plotted with the concentration of larvae extracts along the x-axis and the rate of inhibition along y-axis. The concentrations of larvae extracts corresponding to 50% inhibition of cell growth was used as work concentrations in later studies. All assays were performed in triplicate.

### Influence of larvae extracts on the permeability of the BBB cell model: Four-hour percolation experiment

After the BBB cell model was constructed, 30 µg/ml larvae extracts were added to the culture fluid in the donor tank. The cells were incubated in a CO_2_ incubator for 12 hours. Then culture fluid was added to the donor tank until it was full and to the receiving tank until the fluid level reached 0.5 cm below that of the donor tank. Notes were taken every 15 minutes to determine how long the fluid level in the donor tank remained high. The fluid level started to drop within 4 hours. BBB cell models without larvae extracts were used as negative controls. Transwell inserts without cells were used as blank controls. For each sample, three duplication wells were set. All assays were performed in triplicate.

### Influence of larvae extracts on the permeability of the BBB cell model: Permeability experiment

After the BBB cell model was constructed, 30 µg/ml larvae extracts were added to the culture fluid of the donor tank. The cells were incubated in a CO_2_ incubator for 12 hours. Then 650 µl culture fluid containing 0.002 mg/ml horseradish peroxidase (HRP, 44 kDa, Sigma) was added to the donor tank and 850 µl culture fluid was added to the receiving tank so that the fluid levels of the two tanks were even. This was in order to eliminate the influence of static pressure on permeability caused by differences in fluid level. Samples 50 µl in volume were taken from the donor and receiver tanks at 0.5, 1, 2, 3, 4, and 8 hours, respectively. Then 50 µl of fresh culture fluid was added to each tank. The collected samples were placed on ELISA plates and kept in a refrigerator at 4°C. Substrates were added for a 5-minute color reaction. The *A*_450_ value was measured after the reaction at 450 nm. HRP permeability was calculated. BBB cell models without larvae extracts were used as negative controls. For each sample, six duplication wells were set. All assays were performed in triplicate. HRP permeability was calculated according to the following formula:
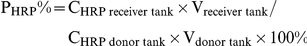



### Detection of cell apoptosis: TUNEL detection

Cell climbing sheets were made using the regular method. Based on the results of experiment 2.3.1, 30 µg/ml larvae extract were added to BMECs and 60 µg/mall to BACs. The cells were incubated for 12 hours. A DeadEnd™ Colorimetric TUNEL System Detection Kit (Promega, Madison, WI, U.S.) was used for TUNEL detection according to the manufacturer's instructions. The results were observed under a high-power lens. All assays were performed in triplicate.

### Detection of cell apoptosis: Annexin-v-FITC/PI double staining detection

A cell climbing sheet was made using regular methods. Based on the results of 2.3.1, 30 µg/ml larvae extracts were added to BMECs and 60 µg/mall to BACs. The cells were incubated for 12 hours. At the end of the incubation, the cells were washed twice with PBS, and 5 µl annexin-V-FITC (Invitrogen, San Diego, CA, U.S.) and 5 µl PI (Invitrogen) were mixed with 500 µl binding buffer. The resulting mixture was added to the surface of the cell climbing sheet and allowed to react in the dark at room temperature for 5 minutes. A laser confocal microscope (Olympus Optical; Tokyo, Japan) was used to observe the results. All assays were performed in triplicate.

### Statistical analysis

These results are expressed as the mean ± SD of three independent experiments. Data from these experiments were analyzed by Student's *t* test (SPSS15.0 software). Significant differences were established at *P*<0.05.

## Results

### Determination of the action concentration of the larvae extracts

An inhibition rate curve was plotted with larvae extract concentrations along the x-axis and the inhibition rate along the y-axis. Cell growth was inhibited by 48% when BMECs were exposed to 30 µg/ml larvae extract. Cell growth was inhibited by 52% when BACs were exposed to 60 µg/ml larvae extract. We then chose to add 30 µg/ml larvae extract to BMECs and 60 µg/ml to BACs during subsequent experiments.

### Influence of larvae extracts on the permeability of the BBB model

**Figure 1:**
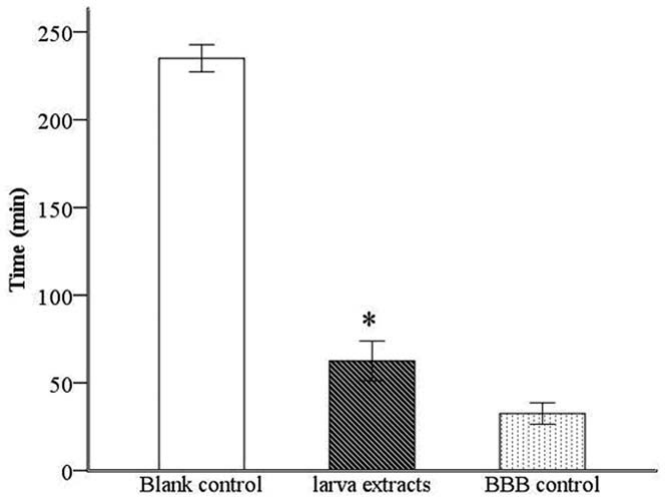
Analysis of percolation ability of BBB model with or without *A. cantonesis* larvae extracts. After larvae extracts were added to the BBB cell model, the time that the fluid level in the donor tank of Transwell started to drop was reported within 4 hours. The fluid level began to decrease significantly sooner in the larvae extract group. Data are presented as the mean ± SD of three experiments. * *P*<0.01, larvae extract group vs. Control group.

A four-hour percolation experiment showed that the fluid level in the donor tank of the larvae extract group remained high for 62.50±11.29 minutes. In the negative control group, it remained high for 235.00±7.75 minutes. The fluid level began to decrease significantly sooner in the experimental group (*P*<0.01) (Fig. 1).

**Figure 2:**
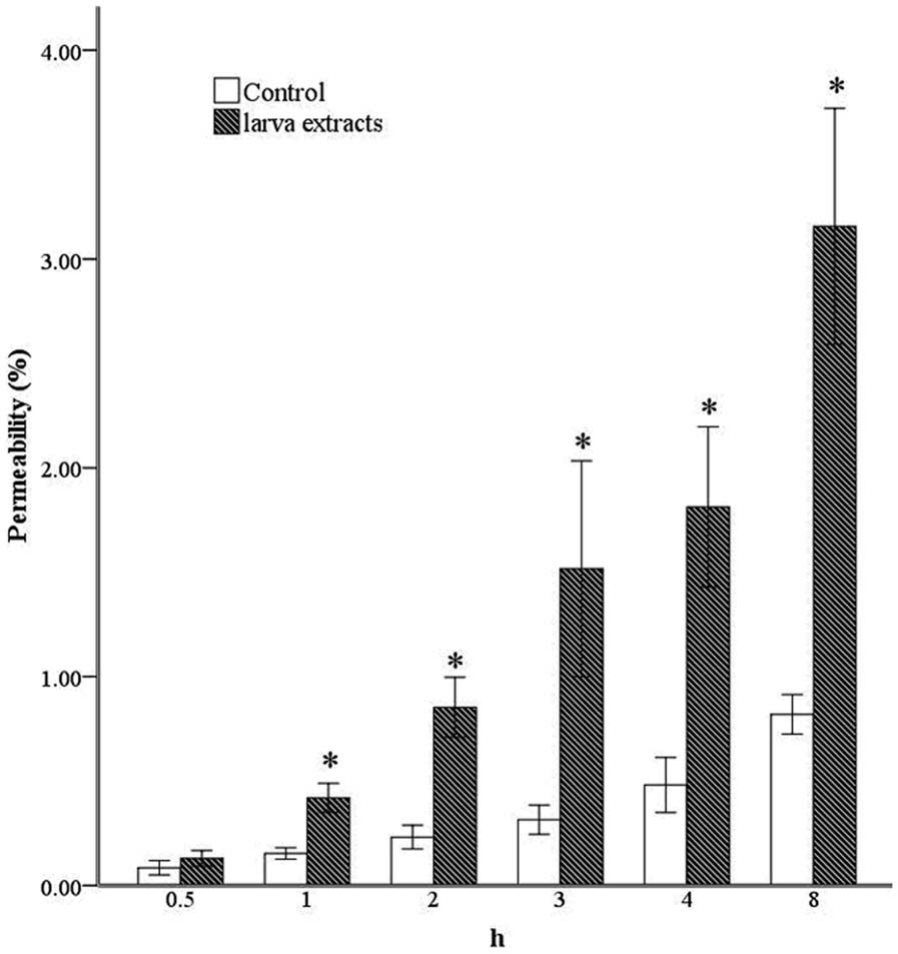
Analysis on HRP Permeability of BBB model with or with *A. cantonesis* larvae extracts. Samples were collected from the donor and receiver tanks to calculate HRP permeability at 0.5, 1, 2, 3, 4, 8 h after larvae extracts were added to the BBB cell model, showing that the HRP permeability started to increase significantly in larvae extract group after 1 hour. Data are presented as the mean ± SD of three experiments. * *P*<0.01, larvae extract group vs. control group at each point in time.

In the HRP permeability experiment, larvae extract was added to the BBB cell model, and samples were collected from the donor and receiver tanks at designated times. HRP permeability was calculated. As shown in Fig. 2, after 1 hour, the permeability of the larvae extract group was 0.42±0.07, significantly higher than that of the control group (0.15±0.03, *P*<0.01). After 4 hours, the permeability of the larvae extract group was 1.81±0.38, and that of the control group was 0.48±0.13 (*P*<0.01), suggesting that *A. cantonensis* larvae extract could induce a 3.77-fold increase in the permeability in this BBB cell model.

### Detection of cell apoptosis

**Figure 3:**
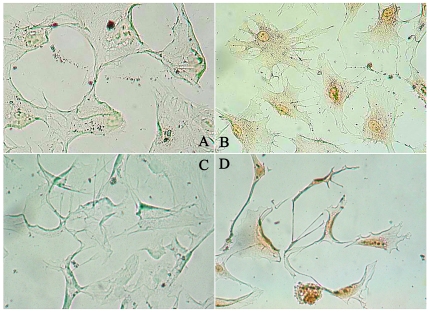
TUNEL detection of apoptosis in BMECs and BACs with and without *A. cantonesis* larvae extracts. No positive signal was detected in (A) BMECs cultured alone or (C) BACs cultured alone. After the addition with larvae extract, positive signals were observed in both (B) BMECs and (D) BACs. This figure shows a representative example of three independent experiments that all gave similar results.

The TdT-mediated dUTP nick end labeling (TUNEL) detection of cell apoptosis was performed 12 hours after the addition of larvae extracts to BMEC and BAC cultures. As shown in Fig. 3, the nuclei of both BMECs and BACs were stained dark brown, suggesting that larvae extract could induce apoptosis in both types of cells.

**Figure 4:**
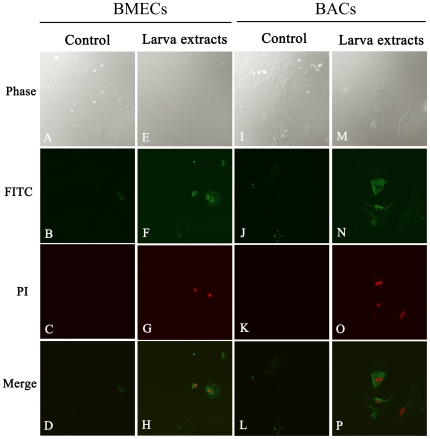
Confocal immunofluorescence images of annexin-V-FITC/PI double staining during *in vitro* culture with or without *A. cantonensis* larvae extracts. No positive signal was detected in alone (A–D) cultured BMECs or (I–L) cultured BACs. With larvae extract, however, positive signals were observed in (E–H) BMECs and (M–P) BACs. The same microscopic field was observed by (A, E, I, M) differential interference contrast, (B, E, H and K) FITC channel, and (C, G, K, O) PI channel. This figure shows a representative example of three independent experiments that all gave similar results.

To avoid false positive results, annexin-V-FITC/PI double staining was used to monitor the progress of apoptosis in BMECs and BACs after the addition of larvae extracts. Annexin-V-FITC staining targets the membranes of apoptotic cells, showing green fluorescence, while PI staining targets the nuclei of apoptotic cells, showing red fluorescence. As shown in Fig. 4, BMECs and BACs that had been exposed to larvae extracts showed fluorescent green cell membranes and fluorescent red nuclei. This also demonstrated that larvae extract could cause apoptosis in both BMECs and BACs.

## Discussion

BBB dysfunction may be involved in the pathological changes associated with angiostrongyliasis. For example, the change in intrathecal immune response in humans can occur as late as 10 days after inflammation [14]. In addition, patients with eosinophilic meningoencephalitis also show increased total protein and albumin levels in the CSF [15] and high CSF albumin to serum albumin ratio that characterizes the neuro-immunological response and the BBB barrier dysfunction [14]. However, due to poor access to significant quantities and dynamic nature of changes of BBB, there is a paucity of information on how *A. cantonensis* larvae enter the brain and how they interact with BBB tissue to induce significant BBB dysfunction. Moreover, this *in vitro* model of the BBB can reduce the need for animal experiments and provide information about the study of these mechanisms [16].

In the present study, therefore, the in vitro mouse model of the BBB was established to determine the pathogenesis of brain injury induced by *A. cantonensis* infection. The structure of the BBB consists of endothelial cells, pericytes, astrocytes, and the vascular basement membrane [17]. The basement membrane can provide the stuctural support to the endothelial cell wall [18], [19]. Thus, using the Transwell filter as a substitute for the BBB's natural vascular basement membrane, the *in vitro* BBB model described in this study consisted of primary culture BMECs and BACs grown opposed to the BMECs on the Transwell filter.

BBB permeability is an important determinant of the BBB function. To determine whether *A. cantonensis* larvae can induce significant BBB dysfunction, this established *in vitro* BBB model was used to assess functional changes in permeability by evaluating percolation over 4 h and HRP permeability after exposure to *A. cantonensis* larvae extract in an activated BBB model. In the 4 h percolation experiments, it was found that the fluid level in the donor tank of the larvae extract group began to decrease significantly sooner than that of the negative control group, indicating that *A. cantonensis* larvae extracts could increase the permeability of the BBB. This deduction was further supported by an increase in the rate of HRP penetration across the BBB in the larvae extract group. These results suggest that some factors in larvae extracts may affect the permeability of the BBB.

To detect how *A. cantonensis* larvae interact with BBB tissue to induce significant BBB dysfunction, we studied the ability of these larvae to induce apoptosis in cultured mice BMECs and BACs. In our previous study, TUNEL assays in brain slices of mice infected with *A. cantonensis* showed that extensive apoptosis (>50% of cells affected), mainly in the cerebella and brain stems of treated mice. Another previous study found that *Plasmodium falciparum* extracts could induce apoptosis in mononuclear cell cultures [20], [21]. Girard *et al.* found that endothelial cell apoptosis could cause the BBB lesions in African trypanosomiasis [22]. In this study, the TUNEL assay and double staining detection revealed that soluble factors from *A. cantonensis* larvae extracts could induce apoptosis in both BMECs and BACs.

Taken together, the results of these studies suggest that larvae of *A. cantonensis* may secrete or shed relevant apoptotic factors into the host bloodstream. These factors can directly affect the BBB dysfunction associated with severe forms of parasitic infection. Several recent studies also support the hypothesis that angiostrongyliasis and other parasitic diseases, such as sleeping sickness (*Trypanosoma brucei* ssp.), cerebral malaria (*Plasmodium falciparum*), toxoplasmosis (*Toxoplasma gondii*), leishmaniasis (*Leishmania* sp.), Chagas disease (*T. cruzi*), and schistosomiasis (*Schistosoma mansoni*) take advantage of apoptosis-mediated pathologies in host tissues [23]–[28]. These studies show that severe inflammation in the CNS, which can be caused by parasitic invasion, is exacerbated by factors secreted directly into the bloodstream, and then these factors enter the CSF through a complex relay of parasite factors and host pro-inflammatory immune modulators. These interactions alter the BBB, rendering it more permeable to cytokines and parasite antigens, which then enter the CSF and the brain compartment [29]. This can cause microglia activation and astrocyte damage, which has also been observed in murine and human cerebral malaria [30]–[33]. The astrocytes are critical to the formation of the BBB in that they regulate endothelial differentiation and increase the expression of BBB-related proteins and enzymes [34], [35]. Also, astroctyes synthesize neuroprotective molecules, so damaging the astrocytes or limiting their ability to perform their functions can disrupt of neuronal activity [29]. Here, we hypothesized that increased expression and secretion of factors from *A. cantonensis* larvae might contribute to BMEC and BAC activation and apoptosis, which perturbs the BBB and can directly or indirectly induce neuroglial apoptosis. Studies are under way to characterize the apoptotic factors and the mechanisms relevant to this pathway. Interestingly, in the current study, the concentrations of larvae extracts found to induce apoptosis in BMECs and ACs were 30 µg/ml and 60 µg/ml, respectively. This suggests that the BMECs used in this study were more susceptible to apoptosis than BACs were. In other words, we have shown that different types of cells have different levels of susceptibility to the apoptotic factors secreted by parasitic larvae. It also seems that during *A. cantonensis* infection, larvae may cause amplified local apoptotic effects in the brain that can result in BBB dysfunction.

In conclusion, the *in vitro* model developed in our laboratory and used in the present study constitutes a practical powerful platform suitable to investigation into the subtle mechanical changes experienced by BBB cells during *A. cantonensis* infection. Although the precise mechanism by which apoptosis is induced under these circumstances and what role it plays in the impairment of the BBB remains to be determined, the present study demonstrates that *A. cantonensis* larvae extracts do indeed have apoptogenic effects in BMECs and BACs. An improved understanding of how *A. cantonensis* induces apoptosis may lead to new approaches in the treatment and prevention of this parasitic infection.
